# Crop and medicinal plants proteomics in response to salt stress

**DOI:** 10.3389/fpls.2013.00008

**Published:** 2013-01-31

**Authors:** Keyvan Aghaei, Setsuko Komatsu

**Affiliations:** ^1^Department of Biology, Faculty of Sciences, The University of ZanjanZanjan, Iran; ^2^National Institute of Crop Science, National Agriculture and Food Research OrganizationTsukuba, Japan

**Keywords:** crops, medicinal plants, proteomics, salt-responsive proteins, secondary metabolites

## Abstract

Increasing of world population marks a serious need to create new crop cultivars and medicinal plants with high growth and production at any environmental situations. Among the environmental unfavorable conditions, salinity is the most widespread in the world. Crop production and growth severely decreases under salt stress; however, some crop cultivars show significant tolerance against the negative effects of salinity. Among salt stress responses of crops, proteomic responses play a pivotal role in their ability to cope with it and have become the main center of notification. Many physiological responses are detectable in terms of protein increase and decrease even before physiological responses take place. Thus proteomic approach makes a short cut in the way of inferring how crops response to salt stress. Nowadays many salt-responsive proteins such as heat shock proteins, pathogen-related proteins, protein kinases, ascorbate peroxidase, osmotin, ornithine decarboxylase, and some transcription factors, have been detected in some major crops which are thought to give them the ability of withstanding against salt stress. Proteomic analysis of medicinal plants also revealed that alkaloid biosynthesis related proteins such as tryptophan synthase, codeinone reductase, strictosidine synthase, and 12-oxophytodienoate reductase might have major role in production of secondary metabolites. In this review we are comparing some different or similar proteomic responses of several crops and medicinal plants to salt stress and discuss about the future prospects.

## INTRODUCTION

Salt stress limits agricultural production throughout the world and is becoming an increasingly global problem which affects about 20% of global irrigated land (Flowers and Yeo, 1995). Many major crops such as pepper, eggplant, potato, lettuce, and cabbage are salt-sensitive (Shannon and Grieve, 1999). In addition, important cereals such as rice and maize are also sensitive to hyperosmotic stresses and their production seriously decreases in saline soils (Ngara et al., 2012). Therefore increasing of soil salinization as well as the growing world population shows the increasing need to develop crops which are able to adapt to salt stress.

Tolerance against salt stress needs profound changes in gene expression which is accompanied with changes in composition of plant transcriptome, metabolome, and proteome. Changes in gene expression at transcript level cannot exactly show the changes at protein level. This reflects the high importance of plant proteome since proteins are directly involved in plant stress response. In addition to enzymes, proteins include components of transcription and translation machinery therefore they can regulate plant stress response at transcript and protein levels (Kosova et al., 2011). Thus, investigation of plant response to stress conditions at protein level can provide a powerful tool to reveal the physiological mechanisms underlying plant stress tolerance.

One of the most popular methods to study of the plant responses to environmental stresses is proteome analysis since the extraction of proteins is easy and the obtained two-dimensional electrophoresis gels have great reproducibility and also the mass spectrometry (MS) for sequencing of proteins are very sensitive (Komatsu et al., 2003). Proteomics has been used to study of the expression of salt stress related proteins in several crops such as rice (Parker et al., 2006), potato (Aghaei et al., 2008a), soybean (Aghaei et al., 2008b), and *foxtail millet* (Veeranagamallaiah et al., 2008), which can provide a better indication of cellular activities under salt stress. According to previous studies stress proteins such as osmotin (Qureshi et al., 2007), reactive oxygen species (ROS) scavenging enzymes (Abbasi and Komatsu, 2004), and pathogen-related proteins (PRPs; Dani et al., 2005) might be used as important molecular markers for the improvement of salt tolerance. Zhang et al. (2012) have summarized 2171 salt-responsive proteins from proteomic analysis of 34 different plant species (19 crops and other plants) and functionally categorized these proteins as follows; photosynthesis, carbohydrate and energy metabolism, metabolism, stress and defense, transcription, protein synthesis, protein folding and transport, protein degradation, signaling, membrane and transport, cell structure, cell division/differentiation and fate, miscellaneous, and unknown function. We have discussed the proteomic responses of 13 economically important crop plants to salt stress and also in compare to five medicinal plants.

Medicinal plants are among the major and important group of crops (Rehm and Espig, 1991) which have been used for traditional prevention and treatment of diseases and using herbal medicines have a long history (Williamson, 2003). Based on the World Health Organization (WHO), about 80% of the world populations still rely on medicinal herbs. Herbal medicinal products are used by nearly 19% of the adult populations in the United States (Kennedy, 2005; Patwardhan et al., 2005); however, the quality and the quantity of secondary metabolites of medicinal plants strongly depend on environmental conditions.

Although the effects of salt stress on crops have been investigated widely and well studied, however, in the case of medicinal plants there is lack of information. The molecular mechanisms of salt tolerance and secondary metabolism in these commercially important crops have not been investigated as other crops. Thus comparing the responses of medicinal plants to salt stress with some other important crops has a great value.

Salt stress significantly affects the production of essential oils and the constituents of medicinal plants, thus the investigation on the mechanisms of salt tolerance in medicinal plants has a great importance. Creation of salt-tolerant medicinal plant leads to increased production of raw materials for drugs, flavors, fragrances, and spices all over the world. In order to increase the production of a special compound in a medicinal plant it is necessary to know which protein or proteins are involved in the biosynthetic pathway and as a consequence, the proteomic approach is a powerful tool to determine the responsible proteins in secondary metabolism in medicinal plants.

Proteomics is also widely used to analyze biochemical pathways and the complex responses of plants to abiotic stresses. Using comparative proteomic investigations of plants before and after specific stresses we are able to reveal the defensive mechanisms which are applied by plants (Timperio et al., 2008). Because of the importance of salt stress effects on crops and medicinal plants and the ability of proteomics as a widely used and powerful tool to determine the proteins which are likely responsible in salt stress responses of these plants in this review we outline the proteome analysis of crops and medicinal plants under salt stress.

## EFFECTS OF SALT STRESS ON CROPS

Salt stress results in impairments in growth of all plants as well as crops, and its effects can be categorized in two short and long terms. Osmotic stress, lowering of external water potential and reduction of water uptake by plants, similar to the situation under drought, all fall in the category of short term effects (Wang et al., 2003); however, ion toxicity occurring when crops are not able to compartmentalize ions properly, falls in the long term effects. In salt-tolerant crops leaf cells can remove Na^+^ as well as Cl^-^ from the cytoplasm and then sequester them in the vacuole. Na^+^ and Cl^-^ are easily taken up when salt concentration in the soil increases which consequently leads to displacement of mineral nutrients such as K^+^, Ca^+^^+^, and also nitrate, which can negatively affect the survival of crops. Introduction of ion excess into the cells causes formation of ROS such as superoxide, hydrogen peroxide, singlet oxygen and hydroxyl radicals, which can disrupt cellular homeostasis and trigger the expression of genes involved in defense mechanisms (Timperio et al., 2008; Du et al., 2010). In addition, gene expression is also influenced by salt stress. For example genes involved in the metabolic pathways of nitrogen reduction and fixation and methionine biosynthesis are significantly affected by salt stress (Ouyang et al., 2007). At higher levels of salt stress, Na^+^ and Cl^-^ have direct toxic effects on the structure of membrane and enzyme leading to the uncontrolled entrance or efflux of minerals and nutrients (Qureshi et al., 2007).

To reveal the facts concerning the proper function of genes and the biochemical kinetics of plants against salt stress, genome sequence information alone is insufficient and consequently it is not possible to determine the exact responsive mechanisms. To solve these problems, more comprehensive approaches including, quantitative and qualitative analysis of gene expression products are necessary at the transcriptome, metabolome, and proteome levels. Many investigations have shown that, some environmental stresses which cause cellular dehydration, like freezing, salt and water stress, often lead to similar changes in plant gene expression and metabolism (Rabbani et al., 2003) and a strong cross-talk can be seen in their signaling pathways. By detection of proteins which are involved in salt stress responses the designated defense mechanism can be inferred (Timperio et al., 2008).

## HOW CROPS RESPOND TO SALT STRESS

Responses of crops to salt stress closely depend on their ability of stress tolerance or sensitivity. Classification of crops, based on salt tolerance showed that; sugar beet and durum wheat are salt-tolerant, broad bean, maize, potato, sunflower, and tomato are regarded as moderately salt-sensitive (Katerji et al., 2000). The classification of soybean in two different classes as moderately salt-sensitive and moderately salt-tolerant can be ascribed to difference in variety (Katerji et al., 2000).

Increasing of the concentrations of NaCl in soil results in osmotic stress which leads to create a misbalance in intracellular ion homeostasis (Chinnusamy et al., 2005). As a consequence of osmotic stress the osmotic adjustment of cell cytoplasm will be induced which leads to accumulation of several low-molecular osmolytes such as; raffinose, glycine betaine, and proline as well as high molecular hydrophilic proteins from late embryogenesis abundant proteins superfamily (Kosova et al., 2011). Glycerol, sucrose, polyamines, putrescine, and other molecules which are not highly charged but polar and highly soluble, have a larger hydration shell to protect biological macromolecules against the damaging effects of salt stress (Sairam and Tyagi, 2004). Accumulation of compatible solutes in the plant tissue is a strategy to combat salinity in sugar beet (Wakeel et al., 2011) and some other crops (Zhu, 2002). Free proline plays a major role in the adjustment of the osmotic potential in a number of crops such as soybean (Aghaei et al., 2008b), potato (Aghaei et al., 2008a), and tomato (Amini et al., 2007) under hyperosmotic conditions. Proline acts both as a protective agent and a free-radical scavenger therefore, crops may overproduce proline in an attempt to regulate the pH of their cytosol (Razavizadeh et al., 2009).

Expression of biosynthetic enzymes is an important way to counter salt stress by increasing compatible solute production (Sakamoto and Murata, 2000) and the enhancement of Na^+^ export from cells and thereby establishment of homeostasis is the other approach against salinity. Zhu (2001) also showed that over-expression of a plasma membrane Na^+^/H^+^ antiporter confers salt tolerance in *Arabidopsis*. Maintaining low cytosolic salt concentrations in crops can be controlled by the ability to selective ion uptake, ion exclusion, and compartmentalization of Na^+^ in vacuoles (Liu et al., 2012). Excess toxic ions, for example, Na^+^, in the cytosol and a deficiency of essential ions such as K^+^ which is a consequence of ionic stress, disrupts ion homeostasis in plant cells (Zhu, 2002) therefore various ion transporters, pumps, and channels play crucial roles in these processes (Zhu, 2003; Reddy and Reddy, 2004).

Antioxidant enzymes and soluble proteins in the cytoplasm of potato (Aghaei et al., 2009) and cotton (Gossett et al., 1994) are able to protect cells from salt-induced oxidative stress. In addition to above mentioned mechanisms which crops use as a strategy against salt stress it has been shown that in tomato, plant growth regulators such as abscisic acid (ABA) and ethylene, also play a pivotal role in the complicated story of abiotic stress (Ouyang et al., 2007).

## WHICH PROTEINS ARE INVOLVED IN CROPS IN RESPONSE TO SALT STRESS?

Plant cells may alter their gene expression in order to tolerate salt stress which results in an increase, decrease, induction or total suppression of some stress responsive proteins such as malate dehydrogenase (NADP+) and pyruvate phosphate dikinase (Ngara et al., 2012). Different families of proteins which have been newly synthesized, accumulated, or decreased are known to be associated with crops response to salt stress (**Table 1**). These proteins may be involved in signaling, translation, host-defense mechanisms, carbohydrate metabolism, and amino acid metabolism. Other proteins such as antioxidant enzymes and chaperonins may encounter salt stressors directly and other groups of proteins like key enzyme in osmolyte synthesis encounter salt stress indirectly (Wang et al., 2004). For these reasons elucidating the various mechanisms of plant response to salt stress and their roles in acquired stress tolerance in crops is of great importance.

**Table 1:** Identified major proteins increased in crop plants under salt stress using proteomics.

No.	Crop plant	Identified protein	Role of protein in salt tolerance	Reference
1	Carrot	Ornithine decarboxylase	Proline biosynthesis	Bohnert et al. (1995)
2	Rice	ABA-responsive proteins	ABA biosynthesis	Moons et al. (1995)
3	Rice	APX, DHAR, SOD	ROS scavenging	Abbasi and Komatsu (2004)
4	Rice	Sti1(HSPs)	Defense mechanisms	Kurek et al. (2002)
5	Pea	Mn-SOD	ROS scavenging	Qureshi et al. (2007)
6	Pea****	PRPs	Defense mechanisms	Qureshi et al. (2007)
7	Potato	Osmotin-like protein	Osmotic stress tolerance	Aghaei et al. (2008a)
8	Wheat	Glutamine synthase	Protein biosynthesis	Caruso et al. (2008)
9	Wheat	Glycine dehydrogenase	Protein biosynthesis	Caruso et al. (2008)
10	Tobacco	PRPs	Defense mechanisms	Dani et al. (2005)
11	Tobacco	Osmotin	Osmotic stress tolerance	Qureshi et al. (2007)
12	Soybean	LEA proteins	Seed development	Aghaei et al. (2008b)
13	Tomato****	NHX1	Ion transport	Ward et al. (2003)
14	Maize****	NHX1	Ion transport	Neubert et al. (2005)
15	Sorghum	Malate dehydrogenase, APX	ROS scavenging	Ngara et al. (2012)
16	Sugar beet	Osmotin-like protein	Osmotic stress tolerance	Hajheidari et al. (2005)
17	Sugar beet	Glycine decarboxylase	Metabolism	Wakeel et al. (2011)
18	Sugar beet	Ferredoxin-NADP-reductase	Photosynthesis and metabolism	Wakeel et al. (2011)
19	Sugar beet	Aminomethyltransferase	Metabolism	Wakeel et al. (2011)

*DHAR, dehydroascorbate reductase; HSPs, heat shock proteins; PRPs, pathogen-related proteins; LEA, late embryogenesis-abundant; NHX1, Na^+^/H^+^ antiporter; APX, ascorbate peroxidase; SOD, super oxide dismutase; ABA, abscisic acid.*

Over-expression of *ornithine decarboxylase* gene in carrot cells indicated that these cells were significantly more tolerant to salt stress (Bohnert et al., 1995). It was also demonstrated that salt-tolerant varieties of rice showed higher levels of ABA-responsive proteins in their roots (Moons et al., 1995). Because of the important roles of proteins in salt stress tolerance in crops analysis of proteins under salt stress has a great value. Therefore proteome analysis which is an important approach has been recently used to investigate the responses of crops to salt stress as well as many other biotic and abiotic stresses.

In a comparative study of salt-resistant and salt-sensitive wheat genotypes, Wang et al. (2007) identified 23 variety-specific salt-responsive proteins. A major group of proteins which increased under salinity in rice were ROS scavenging enzymes such as: ascorbate peroxidase, dehydroascorbate reductase, peroxiredoxin, superoxide dismutase. Increasing of ROS scavenging enzymes suggests that salt stress results in oxidative stress in rice (Salekdeh et al., 2002; Abbasi and Komatsu, 2004). It has been shown that in pea cultivars exposed to NaCl, leaf mitochondrial Mn-SOD, and chloroplast Cu/Zn-SOD activities increased under salt stress (Hernandez et al., 1995).

Nucleoside diphosphate kinase and guanine nucleotide-binding protein which are involved in nucleotide metabolism and enoyl-ACP reductase involving in fatty acid metabolism in rice have been increased under salt stress (Dooki et al., 2006). GTP-binding protein, lectin-like protein, ribulose-1,5-bisphosphate carboxylase/oxygenase (RuBisCO) activase, ferritin, fructose bisphosphate aldolase, photosystem II oxygen-evolving complex protein, oxygen-evolving enhancer (OEE) protein 2, and SOD were increased in rice (Abbasi and Komatsu, 2004). PRPs, phytochelatins, chaperonins, and metallothioneins were also involved in responses to salt stress in pea (Qureshi et al., 2007). In wheat (*Triticum durum*) it has been shown that, glutamine synthase which is a key enzyme for proline biosynthesis and glycine dehydrogenase which is crucial for glycine betaine biosynthesis were significantly increased under salinity stress (Caruso et al., 2008).

Using proteomic approaches, it has been indicated that chitinases, which are PRPs, were accumulated in leaf apoplast of tobacco plants under saline conditions (Dani et al., 2005). It has been confirmed that in transgenic variety of rice, protein sti1 which is one of the heat shock proteins, appeared to be increased in response to salt stress (Kurek et al., 2002; Qureshi et al., 2007). These proteins are among well known stress responsive proteins which are expressed in response to abiotic stresses such as heat, cold, drought, salinity, and oxidative stress (Wang et al., 2004). Aggregation of stress-denatured proteins is prevented by heat shock proteins, and they facilitate the refolding of proteins in order to restore their native biological functions (Wang et al., 2004).

Oxidative stress tolerance related proteins, glycine betaine synthesis, photosynthesis, adenosine triphosphate (ATP) production, protein degradation, cyanide detoxification, and chaperone activities were increased in *Suaeda aegyptiaca* leaves (Askari et al., 2006). According to Aghaei et al. (2008b) beta-conglycinin and late embryogenesis-abundant protein were increased in soybean root and hypocotyl when exposed to salt stress.

It seems that in transgenic *Arabidopsis* and tomato plants vacuolar Na^+^/H^+^ antiporter could confer salt tolerance (Ward et al., 2003). The over-expression of NHX1 contributed to improve salt-resistance in maize (Neubert et al., 2005). A novel ethylene responsive factor called TERF1 was identified by Huang et al. (2004) in tomato, which might have function as a link between the ethylene and osmotic stress pathways (Huang et al., 2004). Enoyl-CoA hydratase and phosphoglycerate mutase-like protein were increased in tomato under salt stress (Amini et al., 2007). In sorghum leaf, it has been shown that the majority of the identified salt stress responsive proteins (28 spots) were energy related proteins (50.9%). RuBisCO subunits, sedoheptulose bisphosphatase, phosphoribulokinase, ribulose 5-phosphate isomerase, OEE1, malate dehydrogenase, and ascorbate peroxidase showed an increased abundance following salt stress (Ngara et al., 2012). Osmotin is one of the most studied proteins that are accumulated in salt adaptation (Qureshi et al., 2007). The osmotin gene is responsible for osmoprotectant synthesis, the most important of which are proline and glycine betaine (Holmstrom et al., 2000). Using a proteomic approach, Hajheidari et al. (2005) in sugar beet and Aghaei et al. (2008a) in potato found an osmotin-like protein increased in drought and salt conditions. It seems that osmotin like proteins as well as osmotin can confer salt tolerance to these crops in response to osmotic stress resulted from salt stress. Using proteomic approach it has been proposed that, NADP reductase, aminomethyltransferase, and decarboxylase subunit T in sugar beet have been increased in response to salt stress (Wakeel et al., 2011) which suggests that, these proteins can be considered as salt-responsive proteins in this crop. In addition to proteins which have been increased in salt stressed crops, RuBisCO small subunits, phosphor glycerate kinase, which catalyses a reduction step in Calvin cycle, and phosphoribulokinase, which catalyses regeneration of primary CO_2_ acceptor RuBP, were all decreased in salt-treated crops such as soybean (Sobhanian et al., 2010) and *Triticum durum* (Caruso et al., 2008).

Regarding above mentioned literature about different proteins which have been induced under salt stress in different crops it can be suggested that salt-responsive proteins are involved in a variety of metabolic processes, such as scavenging of ROS, signal transduction, transcription and translation, transporting, chaperones, cell wall biosynthesis, photosynthesis, processing and degradation, metabolism of energy, amino acids, and hormones.

## EFFECTS OF SALT STRESS ON MEDICINAL PLANTS

Medicinal and aromatic plants are cultivated because of their active constituents which are used for different purposes, especially for production of anticancer drugs such as vincristine and paclitaxel (Verpoorte et al., 2002). In order to create high yielding genotypes of these plants under different environmental conditions, response of medicinal and aromatic plants to salinity stress has been reported in a substantial number of literatures (Said-Al Ahl and Omer, 2011).

Salt stress affects medicinal plants on different physiological stages. One of the most salt-sensitive growth stages which is severely inhibited with increasing salinity is seed germination stage (Sosa et al., 2005). Seed germination of *Ocimum basilicum* (Miceli et al., 2003), *Petroselinum hortense* (Ramin, 2005), sweet marjoram (Ali et al., 2007), and *Thymus maroccanus* (Belaqziz et al., 2009) showed significant decrease under salt stress. Another stage which is negatively influenced by salinity is seedling growth. It has been previously reported that, seedling growth of *Thymus maroccanus* (Belaqziz et al., 2009), basil (Ramin, 2005), chamomile and marjoram (Ali et al., 2007) were severely decreased under salt stress. Slow or less mobilization of reserve foods, suspending the cell division, enlarging and injuring hypocotyls which are induced by salt stress have been proposed as main reasons for these effects (Said-Al Ahl and Omer, 2011).

Morphological characteristics such as number of leaves, leaf area, and leaf biomass have been reduced under salt stress in a number of medicinal plants. Similarly, negative effects of salt stress have been observed in *Majorana hortensis* (Shalan et al., 2006), peppermint (Aziz et al., 2008), geranium (Leithy et al., 2009), *Thymus vulgaris* (Najafian et al., 2009), sage (Ben Taarit et al., 2009), and *Mentha pulegium* (Queslati et al., 2010). Increasing salt stress pronouncedly increased total soluble carbohydrate contents of *Salvia officinalis* (Hendawy and Khalid, 2005) and *Satureja hortensis* (Najafi et al., 2010).

There are some common responses between medicinal plants and crops in response to salt stress and the mechanisms of salt tolerance in medicinal plants include many of the mechanisms which have been explained for crops. Synthesis of compatible solutes such as proline (Ghoulam et al., 2002), nitrogen-containing compounds such as amino acids, amides, proteins, polyamines (Mansour, 2000), and soluble sugars such as glucose, fructose, sucrose, and fructans, have been reported in medicinal plants under salt stress (Omami et al., 2006). It is supposed that these compounds function in protection of cellular macromolecules, scavenging of free radicals, osmotic adjustment, storage of nitrogen, and maintenance of cellular pH (Said-Al Ahl and Omer, 2011). However, because of the presence of high concentrations of different aromatic compounds in these plants such as isoprenoids, phenols, or alkaloids, at least parts of their response to salt stress is mediated by antioxidant properties of these aromatic compounds. Phenylpropanoid derived phenols such as flavonoids, tannins, and hydroxycinnamate esters, which are produced in the course of various stress situations, represent important radical scavengers (Selmar, 2008).

## SALT STRESS AND SECONDARY METABOLISM IN MEDICINAL PLANTS

Secondary metabolites are supposed to have several functions, e.g., protective role against herbivores or pathogens, or an attracting role for pollinators and seed spreading animals in medicinal plants (Selmar, 2008). The close relationship between plant secondary metabolism and defense response is widely recognized (Vasconsuelo and Boland, 2007). Alkaloids, anthocyanins, flavonoids, quinones, lignans, steroids, and terpenoids are among plant secondary metabolites which have found commercial applications as drug, dye, flavor, fragrance, insecticide, and antioxidants (Jacobs et al., 2000; Verpoorte et al., 2002). Therefore secondary metabolites of medicinal plants have a great value; however, molecular mechanisms underlying their production have not been investigated widely.

Production of secondary metabolites by plants is not always satisfactory and there are several factors which can restrict their production. Type of medicinal plant species or genus, particular growth or developmental stage, specific seasonal conditions, nutrient availability, or stress conditions are among these factors (Verpoorte et al., 2002). It has been shown that reactions to salt and drought stress might be responsible for the increase or decrease in the content of relevant natural products; however, scientific background in this field is still rare (Selmar, 2008). Medicinal plants under salt stress similar to drought conditions accumulate higher concentrations of secondary compounds than control plants which are cultivated under standard conditions. Selmar (2008) monitored a strong increase in the concentration of tropane alkaloids in *Datura inoxia* plants under salt stress.

Essential oils are among important secondary metabolites in medicinal plants. Contradictory reports have been found concerning the response of medicinal plants in terms of essential oil production to salt stress. Essential oil yield decreased under salt stress in *Trachyspermum ammi* (Ashraf and Orooj, 2006). Similar results have been reported in several medicinal plants, e.g., *Mentha piperita* (Tabatabaie and Nazari, 2007), *Thymus maroccanus* (Belaqziz et al., 2009), basil (Said-Al Ahl and Omer, 2011), and apple mint (Aziz et al., 2008). Although the main essential oil constituents in *Matricaria recutita* increased under salt stress (Baghalian et al., 2008); however, in *Origanum vulgare* the content of carvacrol (the main essential oil constituent) and p-cymene and γ-terpinene decreased under salt stress (Said-Al Ahl and Omer, 2011). Phenolic acid concentration increased in spearmint (Al-Amier and Craker, 2007), *Achillea fragrantissima* (Abd EL-Azim and Ahmed, 2009), and *Mentha pulegium* (Queslati et al., 2010) as a result of salt stress. These contradictory results represents the different responses of medicinal plants to salt stress regarding to their essential oil production; however, the fact that, some medicinal plants may increase the production of essential oil concentration or its main constituents in response to salt stress encourage us to determine the molecular mechanisms of salt stress on the production of secondary metabolites in medicinal plants.

## PROTEOMIC ANALYSIS OF MEDICINAL PLANTS TO INVESTIGATE THE PRODUCTION OF SECONDARY METABOLITES

Technical developments in genomics, proteomics, and metabolomics represent a challenging complexity for scientific analysis and will open new perspectives for ethno botanical and phytomedical research purposes. Proteomics provides a promising approach for studying secondary metabolism in plants and plant cells. Unfortunately the natural yield of secondary metabolites in medicinal plants is generally low and the biochemistry of the biosynthesis of these compounds is too complicated which is poorly understood. Cell suspension cultures and metabolic engineering are among strategies to increase the yield of these commercially important chemicals. Over-expression of rate limiting enzymes which are involved in biosynthesis of these compounds has been used for this purpose (Verpoorte et al., 1999). Therefore it is necessary to identify proteins involved in the secondary metabolites biosynthesis. Enzyme isolation and characterization by current approaches is time consuming and troublesome, thus, the proteomic approach is a faster and more complete and using this technology we are allowed to identify regulatory and transport proteins as well as enzymes.

**Table 2** summarizes recent proteomic investigations on some medicinal plants and represents some of proteins which are supposed to involve in the production of secondary metabolites. The medicinal plant *Catharanthus roseus *has been used as a best studied model system for secondary metabolite production (Verpoorte et al., 1997). This plant produces some effective anti-tumor drugs, vinblastine and vincristine which are alkaloid compounds. However, alkaloid yields in suspension cell cultures are generally too low to allow commercialization. Jacobs et al. (2000) has used two-dimensional gel electrophoresis for proteomic investigations of alkaloid production in *C. roseus*. Influence of zeatin and 2,4-dichlorophenoxyacetic acid (2,4-D) on protein patterns and alkaloid accumulation of *C. roseus* in a proteomic approach showed that, proteins, which were decreased by 2,4-D and increased by zeatin, may have a direct function as an enzyme or an indirect role as a regulatory or transport protein in alkaloid biosynthesis. A 28 kDa polypeptide which increased by zeatin showed a close correlation with alkaloid production (Jacobs et al., 2000). Another proteome analysis of *C. roseus* resulted in the differential expressions of 88 protein spots which were selected for identification by MS. Full protein spots were identified including two isoforms of strictosidine synthase, which catalyzes the formation of strictosidine in the alkaloid biosynthesis; tryptophan synthase, which is needed for the supply of the alkaloid precursor tryptamine, 12-oxophytodienoate reductase, which is indirectly involved in the alkaloid biosynthesis as it catalyzes the last step in the biosynthesis of the regulator jasmonic acid (Jacobs et al., 2005). Jasmonic acid is essential for signal transduction in the production process of alkaloids. Its involvement in stress reactions was demonstrated by induction of defense proteins including proteins involved in the biosynthesis of secondary metabolites. Jasmonic acid induces alkaloid biosynthesis in *C. roseus* (Jacobs et al., 2005).

**Table 2:** Identified proteins and their role in secondary metabolism in medicinal plants using proteomic approach.

No.	Medicinal plant	Identified protein	Role of protein in secondary metabolism	Reference
1	*Catharanthus roseus*	Strictosidine synthase	Biosynthesis of strictosidine in alkaloid biosynthesis	Jacobs et al. (2005)
2	*Catharanthus roseus*	Tryptophan synthase	Biosynthesis of tryptamine as alkaloid precursor	Jacobs et al. (2005)
3	*Catharanthus roseus*	12-oxophytodienoate reductase	Biosynthesis of the regulator jasmonic acid	Jacobs et al. (2005)
4	*Panax ginseng *	Enolase, glyceraldehyde 3-phosphate dehydrogenase, aldolase	Ginsenoside biosynthesis	Nam et al. (2005)
5	*Chelidonium majus*	Disease/defense-related proteins	Secondary metabolite metabolism	Nawrot et al. (2007)
		Nucleic acid binding proteins		
6	*Papaver somniferum*	Codeinone reductase	Morphine biosynthesis	Decker et al. (2000)

Effects of the secondary metabolites of *Salvia miltiorrhiza* on atherosclerotic lesions (Hung et al., 2010) and cancer have been investigated at the proteomic level (Buriani et al., 2012). Another medicinal plant which has been analyzed by proteomic approach is *Panax ginseng*. The important secondary metabolites of ginseng especially ginsenoids are mainly produced in its roots. In a proteomics experiment in order to find the proteins which are involved in production of its important secondary metabolites, the hairy roots of *Panax ginseng* were been cultured and the root proteome was analyzed. From the 159 cultured hairy root proteins in ginseng, the putative functions of 91 proteins were ascertained. More than 20% of the identified proteins were related to energy metabolism and stress response. Although some proteins such as enolase, glyceraldehyde 3-phosphate dehydrogenase, and aldolase were identified as isotypes; however, none of them was directly related to the metabolism of secondary products (Nam et al., 2005).

In order to determine the major protein content of medicinal plant *Chelidonium majus*, two-dimensional gel electrophoresis of milky sap was investigated. Among 21 protein spots, defense related proteins, nucleic acid binding proteins as well as signaling proteins comprised the main protein components of milky sap in this plant (Nawrot et al., 2007). However, none of identified proteins in this plant could be directly related to its secondary metabolites production. In another attempt, to determine which proteins are involved in production of morphine or other secondary metabolites in opium poppy Decker et al. (2000) performed a proteomic analysis using two-dimensional gel electrophoresis of poppy latex. The main detected protein was codeinone reductase which suggests that codeinone reductase might be a specific enzyme in morphine biosynthesis in this important medicinal plant.

The main limitation for the identification of proteins from medicinal plants is the lack of sequence data of both genes and proteins. For example, the SWISS-PROT database still contains only a few protein entries from *C. roseus* (Jacobs et al., 2005), *Panax ginseng* (Nam et al., 2005), *Papaver somniferum* (Decker et al., 2000), and many other medicinal plants. Consequently the lack of genomic DNA sequence and protein information constitutes a major bottleneck with regard to the identification of proteins in proteomic studies of medicinal plants.

## FUTURE PROSPECTIVE

Among abiotic stresses, salt stress is a well studied stress in which proteomic based techniques have provided a powerful tool to reveal the molecular mechanisms of salt stress responses. Several salt-responsive proteins have been proposed for crops using these techniques and using these proteins and their corresponding genes it will be possible to change salt-sensitive crops to salt-tolerant crops in near future. Despite these advances in crop stress tolerance researches using proteomics, there is still a few and limited investigations in the field of proteome analysis of medicinal plants under salt stress or other situations. Although medicinal plants are very important regarding to their secondary metabolites which are used as herbal drugs, flavors, fragrances, spices, and other purposes, the molecular mechanisms underlying the production of these compounds still remained unclear. There are several pharmaceuticals on the market that are highly expensive due to the fact that these compounds are only found in rare plants and often in extreme low concentrations. Podophyllotoxin (Jusling et al., 2006) and paclitaxel (Verpoorte et al., 2002) are clear examples of pharmaceuticals that can only be produced through the isolation from plants. To achieve a sustainable source of such compounds scientists all over the world have been experimenting with biotechnological approaches aiming at the development of an alternative production system. However, metabolite engineering strategies in this field are time consuming and troublesome.

Proteomics technology has proven its success in many fields of plant sciences, thus in the field of phytochemistry, proteomic approaches have been followed and changes in protein expression could be correlated with the accumulation of secondary metabolites. In spite of a few successes in proteomic analysis of some medicinal plants, the results obtained so far make it clear that proteomics still has to fulfill much of its presumed potential in this area. Although proteomic techniques are rapidly developing; however, some practical constraints still remain which are mainly associated with sample preparation and protein identification. In addition to these problems, one of the major limitations in this field is the lack of genome information about medicinal plants. Many of detected proteins in proteomic analysis of medicinal plants have been remained unidentified. Therefore advances in genome-based information about medicinal plants will lighten new horizons in the field of medicinal plants proteomics. We hope that in near future identification of key proteins involved in the production of secondary metabolites in medicinal plants will leads to increased production of highly appreciated anticancer compounds like vincristine, paclitaxel (Verpoorte et al., 2002), vinblastine (Verpoorte et al., 1997), podophyllotoxin (Jusling et al., 2006), and other valuable herbal drugs which are critical for human beings health all over the world.

## Conflict of Interest Statement

The authors declare that the research was conducted in the absence of any commercial or financial relationships that could be construed as a potential conflict of interest.
